# Assembly of the Complete Sitka Spruce Chloroplast Genome Using 10X Genomics’ GemCode Sequencing Data

**DOI:** 10.1371/journal.pone.0163059

**Published:** 2016-09-15

**Authors:** Lauren Coombe, René L. Warren, Shaun D. Jackman, Chen Yang, Benjamin P. Vandervalk, Richard A. Moore, Stephen Pleasance, Robin J. Coope, Joerg Bohlmann, Robert A. Holt, Steven J. M. Jones, Inanc Birol

**Affiliations:** 1 Canada’s Michael Smith Genome Sciences Centre, British Columbia Cancer Agency, Vancouver, BC, Canada; 2 Michael Smith Laboratories, University of British Columbia, Vancouver, BC, Canada; Montana State University Bozeman, UNITED STATES

## Abstract

The linked read sequencing library preparation platform by 10X Genomics produces barcoded sequencing libraries, which are subsequently sequenced using the Illumina short read sequencing technology. In this new approach, long fragments of DNA are partitioned into separate micro-reactions, where the same index sequence is incorporated into each of the sequencing fragment inserts derived from a given long fragment. In this study, we exploited this property by using reads from index sequences associated with a large number of reads, to assemble the chloroplast genome of the Sitka spruce tree (*Picea sitchensis)*. Here we report on the first Sitka spruce chloroplast genome assembled exclusively from *P*. *sitchensis* genomic libraries prepared using the 10X Genomics protocol. We show that the resulting 124,049 base pair long genome shares high sequence similarity with the related white spruce and Norway spruce chloroplast genomes, but diverges substantially from a previously published *P*. *sitchensis- P*. *thunbergii* chimeric genome. The use of reads from high-frequency indices enabled separation of the nuclear genome reads from that of the chloroplast, which resulted in the simplification of the de Bruijn graphs used at the various stages of assembly.

## Introduction

With the use of short DNA sequence reads continuing to present challenges for *de novo* genome assembly, new technologies in sequencing are offering potential solutions, including the longer read lengths produced by instruments from Pacific Biosciences (Menlo Park, CA) and Oxford Nanopore Technologies (Oxford, UK). More recently, a platform devised by 10X Genomics (Pleasanton, CA) generates barcoded sequencing libraries with long-range linkage information. The GemCode platform from 10X Genomics partitions long pieces of DNA into individual emulsifications containing beads (gems) with attached oligonucleotides [1]. These oligonucleotides include adapter sequences, as well as one of 750,000 possible 14 base pair (bp) index sequences that are incorporated into each Illumina (San Diego, CA) sequencing fragment. As a result, each sequencing fragment from a given partitioned piece of DNA are barcoded with the same index sequence. Therefore, the GemCode platform improves on existing short read sequencing technology, and enables pooling of paired-end sequences by index, thus grouping together sequences that arise from the same original piece of DNA [1]. Because there will be more partitioned pieces of DNA than unique indices for larger genomes, it is expected that some indices are reused. In February 2016, 10X Genomics released the Chromium System, which uses the same approach of partitioning DNA pieces to create barcoded sequencing libraries as the GemCode platform used in this study, but supports nearly four times more barcodes. This feature is expected to allow more specific reads-to-genomic locus assignments as it curbs index re-usability.

Using the GemCode technology, we studied the genome of the Sitka spruce tree (*Picea sitchensis*), an ecologically and economically important conifer species naturally found in coastal areas of North America's Pacific Northwest region [2]. Here, we report on the assembly of the chloroplast genome of the species.

Chloroplasts, the photosynthetic organelles of plant cells, have genomes averaging from 120 to 160 kb long, and have highly conserved regions as well as more variable regions, both of which can convey important information about the evolution of the plant species [3]. Recently, the chloroplast genomes of the white spruce (*Picea glauca*, clone PG29) and Norway spruce (*Picea abies*) trees have been sequenced [4, 5].

Because a single plant cell has many chloroplasts, numerous copies of the chloroplast genome are included in the total genomic sample for sequencing, compared to only one copy of the nuclear genome per cell [6]. In fact, using 3D imaging techniques, Kubinova *et al*. [6] estimated that the mesophyll cells of Norway spruce needles have an average of 209 chloroplasts per cell. Therefore, it is expected that many more sequencing reads will cover the chloroplast genome sequence compared to the nuclear genome. Whereas some groups opt to use DNA purification methods to separate the organellar genomes from the nuclear genome for sequencing [7], our group has recently shown that this can be done informatically using the fact that organelles have high-copy numbers and the DNA derived from them is oversampled [4]. Genome assembly methodologies that exploit this overrepresentation of sequence reads and constituent substrings of length k (k-mers) are amenable to plasmid and organellar genome assembly.

The GemCode library preparation protocol samples long fragments in a non-redundant manner, and has a wide distribution for fragment representation. While some indices have only a few reads associated with them, others may have several thousand reads. This is of course confounded with index reuse. Still, indices with a high number of associated reads would be enriched in chloroplast fragments, as would any arbitrary subset of the sequencing data; but this particular subset would also have the chloroplast sequences co-localized on indexed DNA fragments, a property which we exploit to assemble the Sitka spruce chloroplast genome.

By capitalizing on the indexing information afforded by the GemCode platform to segregate out sequencing reads from indices with a high number of associated reads, and using the previously determined sequence of the related [8] white spruce admix (PG29 genotype) chloroplast genome [4] for scaffolding and read filtering, we were able to assemble the complete chloroplast genome of the Sitka spruce tree.

## Results

DNA was isolated from the needles of a Sitka spruce tree genotype Q903 [2] as previously described [4, 9], and a sequencing library was prepared using 10X Genomics' GemCode platform. The library was sequenced with paired-end (2x125bp) reads using Illumina's HiSeq instrument. To subsample the reads from indices with a large number of associated reads, we determined the number of reads associated with each fully sequenced index (containing no ambiguous, N, bases), then grouped the reads into “bins” (S1 and S2 Figs). These bins included reads from all indices with at least 1,000 (top 33.8% of reads), 3,000 (top 2.4%) and 5,000 (top 0.8%) reads, hereon referred to as 1k, 3k and 5k bins, respectively (S1 Table). The most selective 5k bin included 238 different indices and a total of 2,290,669 read pairs. Since this bin does not exclusively represent the chloroplast genome, it is not possible to say *a priori* what its read abundance translates to in terms of coverage redundancy. However, when aligned (using BWA [10]) to the closely related white spruce chloroplast genome, reads in the 5k bin covered 99.9% of the reference. This demonstrated that this small subset of the entire data likely covers almost all of the Sitka spruce chloroplast genome sequence. Hence our efforts to assemble the Sitka spruce chloroplast genome are based on the 5k bin.

To begin the assembly, the 5k bin reads were assembled using ABySS [11]. The *de novo* assembly resulted in 108 contigs 500 bp or longer in length, 67.6% of which mapped to the chloroplast genome of the related white spruce, as determined through analysis of the contigs using QUAST (S2 Table) [12]. We note that compared to an assembly of all reads, using the reads in the 5k bin resulted in a much simpler de Bruijn graph, measured by the number of blunt ends in the graph: 7.7% blunt ends in the 5k bin assembly, compared to 15% blunt ends in the assembly of the entire read set.

These resulting contigs were scaffolded using LINKS [13], supplying the related white spruce chloroplast genome to order and orient the Sitka spruce contigs. This step produced 34 scaffolds, but only one, a 122,544 bp scaffold with 74 gaps, mapped to the white spruce chloroplast genome (S3 Fig), as determined through analysis of the scaffolds using QUAST (S2 Table). Therefore, all of the chloroplast-derived contigs from our high-frequency index reads co-assembled in this single scaffold.

To finish the genome, we first ran the gap-filling software, Sealer [14], on the identified chloroplast scaffold, supplying the reads from the 3k bin, and using k-mers ranging from 125 to 35 bp, as detailed in Methods. This run closed 68 of the 74 gaps. We note that using this read set achieved higher base coverage of the white spruce chloroplast genome compared to the reads from the 5k bin, as determined through BWA alignments.

A failure-mode analysis of the gap-filling runs revealed that for the lowest k-mer size assessed (k = 35), all of the remaining gaps failed to close either due to the number of branches or the number of paths exceeding our allowed thresholds (B = 3000 and P = 20, respectively) (S3 Table). We reasoned that the base pair information needed to close the gaps was likely present in the provided read subset, but extraneous reads were over-complicating the de Bruijn graph. Therefore, we next used alignments between the remaining gap regions of the Sitka spruce chloroplast scaffold and the white spruce chloroplast genome to filter out reads that would likely span the gap regions. First, local BLAST alignments [15] were performed between the scaffold gaps plus 500 bp of flanking sequences and the white spruce chloroplast genome. Then, for each alignment, the unaligned region plus 24 bp of flanking sequence was extracted from the white spruce chloroplast genome. Because of the noted high sequence similarity between the white spruce and Sitka spruce chloroplast genomes, we expected this extracted region to be highly similar to the corresponding gap region on the Sitka spruce chloroplast scaffold. Using the BioBloomTools utility [16], a Bloom filter was built from the extracted sequence to filter the reads from the 3k bin. This filtering step enriched for reads that could span the gap region on the Sitka spruce chloroplast scaffold, thus simplifying the graph to encompass k-mers from only a local area of the genome. The filtered reads were used in subsequent Sealer runs, closing four of the remaining six gaps on the scaffold. We used a similar strategy to target the last two gaps, utilizing reads from the 1k bin and the entire read set.

Finally, through BLAST alignments of the ends of our Sitka spruce chloroplast scaffold to the white spruce chloroplast sequence, we determined that our scaffold was missing around 40 bp of sequence at the 5' end, and 2 kb of sequence at the 3' end. To recover these missing bases, we used Konnector [17], which fills in nucleotides between paired-end reads given a Bloom filter representation of a de Bruijn graph. Since we expected the chloroplast genome to be circular, we supplied the 5’ and 3’ ends of our scaffold as the “paired-end read” input to Konnector to fill in the missing bases between the two ends. To make the Bloom filter, we followed an approached similar to how we filled in the last two gaps: we filtered reads in the 3k bin associated with the target region of the white spruce chloroplast genome. We then used ABySS-bloom to build a Bloom filter from this read subset. With this Bloom filter, Konnector filled in the missing sequences between these ends, which were then manually appended to the Sitka chloroplast scaffold. We polished the resulting draft genome using the Genome Analysis Toolkit [18], which confirmed all the existing bases.

**Fig 1:**
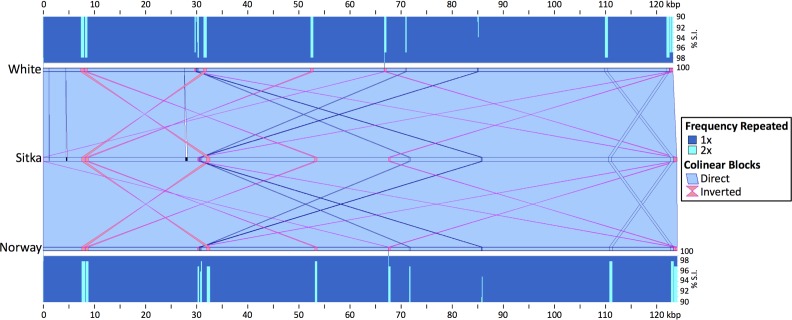
Alignments of the Sitka spruce chloroplast genome to the white spruce and Norway spruce chloroplast genomes. The cross_match alignments were visualized using XMatchView. Histograms at the top and bottom show the sequence identity (S.I.) over the length of the alignments, including those from repeated sequences. The dark blue represents sequences repeated only once, while the light blue represents sequences repeated twice. The middle section represents co-linear and inverted sequence alignment blocks in blue and pink, respectively.

**Fig 2:**
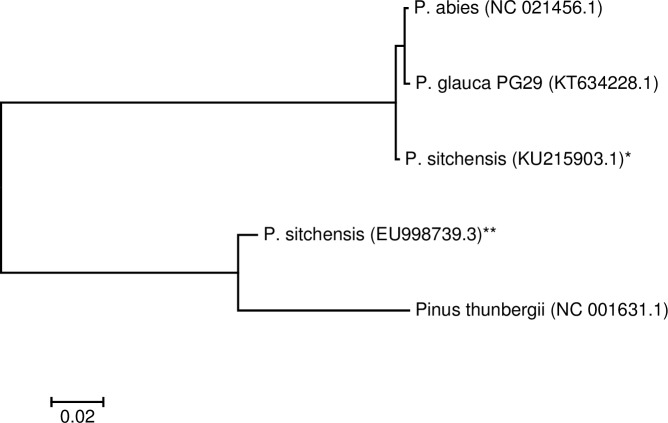
Molecular Phylogenetic analysis of five conifer chloroplast genomes by Maximum Likelihood method. The evolutionary history was inferred by using the Maximum Likelihood method based on the Tamura-Nei model [20]. The tree with the highest log likelihood is shown. Initial tree(s) for the heuristic search were obtained automatically by applying Neighbor-Join and BioNJ algorithms to a matrix of pairwise distances estimated using the Maximum Composite Likelihood (MCL) approach, and then selecting the topology with superior log likelihood value. The tree is drawn to scale, with branch lengths measured in the number of substitutions per site. The analysis involved 5 chloroplast genome nucleotide sequences, white spruce (*Picea glauca* genotype PG29, KT634228.1 [4]), Norway spruce (*P*. *abies* NC021456.1 [5]), Sitka spruce (*P*. *sitchensis* [2] from *our study KU215903.1 and from **previous public genome sequence EU998739.3 [19]), and Japanese black pine (*Pinus thunbergii* NC_001631.1 [21]). Codon positions included were 1st+2nd+3rd+Noncoding. All positions containing gaps and missing data were eliminated. There were a total of 106,346 positions in the final dataset. Evolutionary analyses were conducted in MEGA7 [22].

From comparing whole genomes by sequence alignment, we observe better overall co-linearity and sequence conservation (average 99.0% sequence identity) between the spruce chloroplast genomes that were independently assembled *de novo* (Fig 1). Further, we note that although the published EU998739.3 *P*. *sitchensis* sequence approaches the Japanese black pine chloroplast sequence in total length (120,175 vs. 119,707 bp, respectively) it is ~4 kbp shorter than that of other spruce chloroplast genomes, including the *P*. *sitchensis* sequence we report herein (124,040 bp) and it also comprises 7,647 ambiguous bases (112,528 resolved bases, 93.6% of its reported sequence, S1 File). Overall, nucleotide composition frequencies for Norway, white and Sitka (our study) are consistent (A = 30.8%,C = 18.9%,G = 19.8%,T/U = 30.5%, while the EU998739.3 and Japanese black pine are consistent with one another (A = 30.6%,C = 19.4%,G = 19.3%,T/U = 30.8%, S1 File). A phylogenetic analysis performed using all four spruce choloroplast genomes and the Japanese black pine supports these observations, as EU998739.3 branches with the pine sequence, separately from Norway, Sitka and white spruce (Fig 2). Effectively, the previously reported *P*. *sitchensis* EU998739.3 sequence [19] has better overall co-linearity with the pine chloroplast genome upon which it is derived (S4 Fig).

**Fig 3:**
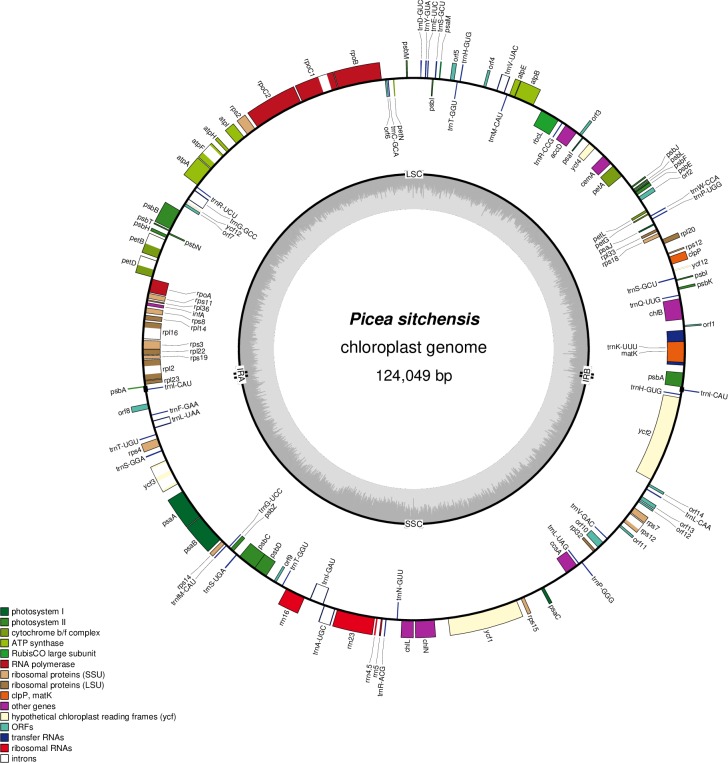
The complete plastid genome of Sitka spruce. The Sitka spruce chloroplast genome was annotated using MAKER and plotted using OrganellarGenomeDRAW [25]. The inner grey track depicts the G+C content of the genome. The genome comprises 74 coding genes, 4 ribosomal RNA (rRNA), 36 transfer RNA (tRNA) genes and 14 ORFs.

Gene annotation of the Sitka spruce chloroplast reveals that all 114 genes are found in the same copy number and in the same order as is observed in white and Norway spruce, including 74 coding genes, 4 ribosomal RNA (rRNA) and 36 transfer RNA (tRNA) genes (Fig 3). The 15 introns seen in white and Norway spruce are also found in Sitka spruce, with 9 found in coding genes and 6 in tRNA. Of these 15 introns, 12 are group II self-splicing ribozymes identified by RNAweasel [23], which are common in plastid genomes. One additional group II intron is found upstream of exon 2 of the trans-spliced gene rps12, consistent with [24], though not included formally in this annotation due to the difficulty in identifying the 5' coordinate of the intron without additional transcript evidence. The three introns not annotated as group II and found in the genes petD, trnL-UAA and trnI-GAU, is either due to a lack of sensitivity in RNAweasel or to a splicing mechanism other than a group II self-splicing ribozyme. We identify 14 open reading frames (ORFs), 13 of which are larger than 150 bp, and 4 of which are larger than 300 bp. Of these 15 ORFs, four (including three ORFs larger than 300 bp) hit genes of other Picea and Pinus species, ndhB, ndhK, rps4 and ycf2, but did not represent the full length of the homologous gene, indicating possible pseudogenes of Sitka spruce. Nine ORFs hit open reading frames and cDNA of Picea and Pinus species, predominantly Korean pine (*Pinus koraiensis*) and Japanese black pine (*Pinus thunbergii*). One ORF of 288 bp has no significant BLASTX hit to the NCBI-nr protein database, but many hits to various conifer genomes in the NCBI-nt nucleotide database (Fig 3 and S4 Table).

The two-copy inverted repeat of Sitka spruce is 440 bp, the same size as Norway spruce, and slightly smaller than the 445 bp inverted repeat of white spruce. The inverted repeat of Norway spruce has perfect sequence identity between the two copies. White spruce observed a single nucleotide mismatch between its two copies. Unusually, Sitka spruce has three nucleotide mismatches between its two copies.

## Methods

### Read Binning Using GemCode Indices

All indices lacking ambiguous bases (‘N’s) were extracted from the sequencing files, and the numbers of associated reads were counted for all extracted indices (S2 Fig). Then, all reads from indices with at least 1,000, 3,000 and 5,000 associated reads were filtered into bins (1k, 3k, and 5k bins, respectively) (S1 Fig). The base coverage of the white spruce admix (PG29 genotype) chloroplast genome [4] by the read sets was determined using BWA-MEM [10] (v0.7.5a, options -k 19) and genomeCoverageBed from BEDTools [26] (v2.25.0, option -d).

### Genome assembly

All reads from the 5k bin were assembled using ABySS [11] (v1.9.0, options -k 48 -l 40 -s 1000 -q 15). K-mer sizes in the dataset are optimized using the contiguity of the resulting assembly, measured by the scaffold N50. The assembly using all reads was run with the same parameters. From these assemblies, blunt ends (i.e. k-mer vertices with no neighbours at least in one direction of sequence extensions) in the de Bruijn graphs were quantified using ABySS-gc. Contigs of length 500 bp or more from the optimal assembly of the 5k bin were scaffolded using LINKS [13], with the white spruce chloroplast genome supplied as “long reads”. LINKS was run iteratively (v1.5.2, options -t 1 -k 26), with distances between the k-mer pairs (-d) ranging from 250 to 16,000 bp (step size 250 for 250–2,000 bp; step size 500 for 2,000–9,000 bp; step size 1,000 for 9,000–16,000 bp). In these iterations, output from the LINKS run with a smaller distance parameter was supplied as input to the LINKS run with the next larger distance specified. The resulting scaffolds were mapped to the white spruce chloroplast genome using QUAST v3.1 [12], yielding a single on-target scaffold (S3 Fig).

### Genome Finishing

Sealer [14] was used for gap-filling, with the post-LINKS scaffolds and reads from the 3k bin supplied as inputs (v1.9.0, options -j 12 -B 3000 -e -b 1200M -F 5000 -P 20, k-mer range from 125–35 (step size 5 for 125–95; step size 1 for 95–35)). Each remaining gap region (and 500 bp of flanking sequence) was aligned to the white spruce chloroplast genome using BLAST [15] (blastall v2.2.22, option -p blastn). For each alignment, the portion of the white spruce chloroplast genome that did not align to the Sitka spruce gap region, plus 24 bp flanking sequence was extracted. The BioBloomTools utility [16] was used to filter out reads that had high similarity to each extracted region. A Bloom filter was made from the extracted sequence using BioBloomMaker (v2.0.12, option -k 25). Then, BioBloomCategorizer used this Bloom filter to filter the reads from the 3k bin (v2.0.12, default options). Next, Sealer was run for each set of filtered reads (S1 Fig, Gap-filling #1 and #2) using the latest Sitka spruce chloroplast scaffold (options -j 12 -B 3000 -e -b 1200M -F 5000 -P 20, k-mer range from 125–35 (step size 5 for 125–95; step size 1 for 95–35)), closing all but two scaffold gaps. To fill the first of the two remaining gaps in the Sitka spruce chloroplast genome, the gap region was aligned to the white spruce chloroplast genome using BLAST (option -p blastn). The region that had no alignment to the Sitka spruce gap region (plus 24 bp of flanking sequence) was extracted from the white spruce chloroplast genome (S1 Fig, Gap-filling #3). BioBloomMaker was used to make a Bloom filter from this extracted region (option -k 25), then the resulting Bloom filter was used with BioBloomCategorizer to filter the reads from the 1k bin (default options). Then, Sealer was run with these filtered reads and the latest Sitka chloroplast scaffold (options -j 12 -B 3000 -e -b 1200M -F 5000 -P 20, k-mer range from 125–35 (step size 5 for 125–95; step size 1 for 95–35)).

To fill the final single 'N' gap, sequences upstream and downstream of the gap were extracted from the Sitka spruce chloroplast scaffold (97 bp upstream of the gap, and 50 bp downstream of the gap), and these sequences were used to make a Bloom filter with BioBloomMaker (options -k 25 -f 0.001). BioBloomCategorizer was then run using this Bloom filter to filter all Sitka reads (options -e -i). Because 73 bp upstream of the 'N' did not align at all to the white spruce chloroplast genome, this region on the Sitka spruce chloroplast scaffold was replaced with 'N's to force Sealer to use a start k-mer upstream of this suspected misassembled region (S1 Fig, Gap-filling #4). Finally, Sealer was run on the latest Sitka chloroplast scaffold using the filtered reads (options -j 12 -B 3000 -e -b 1200M -F 5000 -P 20, k-mer range from 125–35 (step size 5 for 125–95; step size 1 for 95–35)). The final, gap-filled assembly was considered for polishing with the Genome Analysis Toolkit [18] (GATK v2.8-1-g932cd3a) using a variant call format (vcf) file generated using bwa (v0.7.10) and samtools pileup (v0.1.19). We note that no bases were edited from this polishing step.

### Circularizing the chloroplast genome

BLAST (option -p blastn) was used to align 200 bp end regions of the latest Sitka spruce scaffold to the white spruce chloroplast genome. The unaligned regions (plus 24 bp flanking sequence) were extracted from the white spruce chloroplast genome. BioBloomMaker was used to make a Bloom filter from these extracted regions (options -k 25 -f 0.001). Then, BioBloomCategorizer was run to use this Bloom filter to filter the reads in the 3k bin (default options). A Bloom filter was then generated from these filtered reads using ABySS-bloom build (v1.9.0, option -k 80). The ends of the Sitka spruce chloroplast scaffold were formatted as “paired-ends reads” by extracting 200 bp from each end, and reverse-complementing the 5' end region. Konnector [17] was run to fill in the nucleotides between these paired-end reads using the constructed Bloom filter (v1.9.0, options -k 80 -F 3500).

### Whole genome comparison and phylogenetic analysis

Five complete chloroplast genome sequences of white spruce [4] (*Picea glauca* genotype PG29, KT634228.1), Norway spruce [5] (*P*. *abies* NC_021456.1), Sitka spruce (*P*. *sitchensis* genotype Q903 [2] from the current study KU215903.1; and previous public genome sequence EU998739.3 [19]), and Japanese black pine [21] (*Pinus thunbergii* NC_001631.1) were aligned in pairs using an implementation of the Smith-Waterman algorithm (cross_match v1.080721; http://www.phrap.org; options -minmatch 29 –minscore 59 –masklevel 101), and visualized using XMatchView (v0.2; http://www.bcgsc.ca/platform/bioinfo/software/xmatchview; options -m 10 –r 50 –c 50 –l 50) (Fig 1 and S4 Fig).

For the phylogenetic analysis, we first performed multiple sequence alignments of the chloroplast genome sequences described above using clustalw [27] (v1.83 with gap opening and extension penalty of 15 and 6.66 for both the pairwise and multiple alignment stages, DNA weight matrix IUB with transition weight of 0.5) and used the resulting pairwise alignments as input for MEGA7 [22]. The evolutionary history was inferred by using the Maximum Likelihood method based on the Tamura-Nei model [20] where initial trees for the heuristic search are obtained by applying Neighbour-Join and BioNJ algorithms to a matrix of pairwise distances estimated using the Maximum Composite Likelihood approach, and then selecting the topology with superior log likelihood value.

### Genome annotation

We annotated the coding (mRNA) and non-coding (rRNA and tRNA) genes of Sitka spruce (Picea sitchensis, KU215903) using MAKER [28] (v2.31.8; options maker -fix_nucleotides). The gene sequences of Norway spruce [5] (*Picea abies*, NC_021456.1) were used as evidence for MAKER. This automated annotation missed six difficult-to-annotate genes, which we annotated manually, based on visualization of the aligned evidence using IGV 2.3.80 [29]. The gene matK is found inside the intron of trnK-UUU. The genes rpl22 and rps3 overlap, as do psbC and psbD. One copy of the gene psbA is truncated and annotated as a pseudogene. The gene trnI-GAU is not annotated by MAKER for reason unknown. The gene rps12 is trans-spliced [24]. MAKER annotated 12 introns and missed three due to short initial exons of length 6, 8 and 9 bp in the genes petB, petD and rpl16, which we annotated manually as above. We annotated open reading frames using Prodigal 2.6.2 [30] and aligned these sequences to the NCBI-nr protein database using BLASTX [15] to identify homologous proteins. We aligned the complete genomes of white spruce (Picea glauca, KT634228.1) [4] and Norway spruce to Sitka spruce using BWA-MEM [10] (v0.7.15; options -xintractg) to investigate the conservation of chloroplast gene synteny between these three closely related species. We identified the two-copy inverted repeat typical of conifer chloroplast genomes using MUMmer [31] (v3.23; options nucmer -p). The Makefile script to perform these analyses, including the command line parameters used for each program, are available online at https://github.com/sjackman/picea-sitchensis-plastid/blob/1.0.0/Makefile

## Discussion

The final, complete Sitka spruce chloroplast genome is 124,049 bp long, with 38.7% GC content. As seen in alignments between the related Sitka spruce, white spruce and Norway spruce chloroplast genomes (Fig 1), our newly constructed genome has high sequence similarity with both related conifer species, with at least 99.0% sequence identity observed over the full length of the alignments.

The Sitka spruce chloroplast genome introduced herein was also compared to a previously published Sitka spruce chloroplast genome (GenBank accession EU998739.3, [19]). The latter sequence is 120,176 bp long, 3,873 bp shorter than the chloroplast genome we assembled, and 3,908 bp and 3,090 bp shorter than the Norway (*P*. *abies*) and white spruce (*P*. *glauca*) chloroplast genomes, respectively. In addition, while our Sitka spruce chloroplast genome shares over 99.0% sequence identity with the related Norway and white spruce chloroplast genomes. In comparison, the EU998739.3 sequence is only 94.4% identical to our Sitka spruce chloroplast genome over aligned regions, on average (S4 Fig). Further, aligning our Sitka spruce chloroplast genome with the EU998739.3 sequence reveals significant structural rearrangements that are not expected between different genotypes (S4A Fig). Altogether, the higher degree of similarity of our sequence to both the Norway and white spruce chloroplast genomes, the perfect chloroplast gene synteny observed between these 3 species and the fact that chloroplast genomes are expected to be highly conserved over evolutionary time supports the improved accuracy of our Sitka spruce chloroplast genome over the EU998739.3 sequence [32]. Finally, while our chloroplast genome has no gaps, the previously published sequence has 109 gaps (70 bp +/- 340.83 bp) totalling over 7.6 kbp and the available sequence, which includes these ambiguous bases is still approximately 4 kbp shorter, consistent with the chloroplast genome sequence length of Japanese black pine.

The observed structural rearrangements and lower sequence identity of the EU998739.3 sequence compared to our Sitka spruce chloroplast genome is likely due to the procedure employed for the assembly of the former, which used the chloroplast genome of Japanese black pine (*Pinus thunbergii*, GenBank accession NC_001631), a species known to be 6.3% divergent from Sitka spruce [19]. Briefly, Cronn et al. [19] prepared their sequencing libraries using PCR products with primers derived from the chloroplast genomes of *Pinus thunbergii* and *Pinus koraiensis* to enrich for Sitka spruce chloroplast genome fragments. They sequenced these fragments on an Illumina 1G Genome Analyzer sequencer to generate 1,263,800 single-end 36bp reads, and assembled the generated data using Velvet [33]. The resulting contigs were ordered and oriented using alignments to the Japanese black pine, and gaps were filled using sequence from this reference to create a chimeric scaffold. Using the original sequencing reads with the chimeric scaffold as the reference, a reference-guided assembly was performed using RGA [19]. Therefore, *Pinus thunbergii* sequence would still be expected to be in the EU998739.3 sequence, resulting in a chimeric genome, which may explain the 5.6% sequence divergence to our Sitka spruce chloroplast genome assembly.

It has been shown that *P*. *sitchensis* and *P*. *glauca* can hybridize [34]. While we used this information as a rationale for utilizing long-range pairing information from the closely related white spruce chloroplast genome [4, 9], the EU998739.3 assembly used the more distantly related chloroplast from the *Pinus* genus to scaffold, which is more likely to have undergone structural rearrangements compared to genomes of the *Picea* lineage. This is in fact the case as the EU998739.3 genome unexpectedly displays better co-linearity with the more distant black pine chloroplast genome (S4B Fig) compared to that of other spruce species (S4A Fig). This observation can also be made from phylogenetic analysis, where the EU998739.3 separates from the other spruce chloroplast sequences to form a separate branch with the more evolutionarily distant pine sequence upon which the chimeric EU998739.3 sequence is derived. This contrasts with all three independent *de novo* assemblies of white, Norway and Sitka spruce chloroplasts ([4, 5] and this study), where conservation between chloroplast sequences of the same genus branches from that of pine, the latter estimated to have diverged from spruce over 100 MYA [35].

By using the GemCode index sequences associated with each sequencing pair, we were able to take smaller samples of the entire read set, and the resulting targeted assemblies resulted in better contiguity and a less complicated de Bruijn graph structure, when compared to both the full read set and the lower frequency index bins. Because many of the steps in our assembly pipeline used graphs, reducing the number of reads while still maintaining high base coverage of the chloroplast genome decreased the complexity and number of paths represented in those k-mer graphs. Therefore, taking advantage of the information available to us from the index sequences led to better results in the initial assembly, gap filling and end extension steps of our Sitka spruce chloroplast genome assembly. In this paper, we report on one of the first *de novo* assembly application of GemCode indexed reads from 10X Genomics, exploiting both the properties of these reads and the elevated copy number of organelles in plant cells by limiting the genome assembly to reads from high-frequency indices exclusively. Using the GemCode platform, genomic fragments are tagged with ~700,000 barcodes or sequence indices, which limits unique assignments of reads to a given genomic fragment/locus. The recently announced Chromium processor from the same vendor increases the number of partitions further by using ~4M barcodes, which is expected to yield sequence reads with increased resolution, making it potentially easier, to characterize a specific locus or target sequence such as that of plasmid and organelle genomes.

### Nucleotide sequence accession number

The Sitka spruce chloroplast genome sequence is available at NCBI GenBank under accession number KU215903. The Illumina/10X Genomics GemCode indexed reads are available from the SRA under accession number SRP068431.

## Supporting Information

S1 Fig Sitka spruce chloroplast genome assembly and finishing strategy. High-frequency GemCode read bins (> = 5k) were assembled with ABySS [11], contigs scaffolded with LINKS [13] and assembly gaps filled with Sealer [14] until all unknown bases (Ns) resolved. The genome completed by extending the ends with Konnector [17].
(PDF)

S2 Fig Distribution of GemCode index multiplicity. Indices are in ascending order with respect to the number of associated reads, and only fully sequenced indices (containing no ambiguous bases) are included.
(PDF)

S3 Fig Dot plot of alignment between the post-LINKS Sitka spruce chloroplast scaffolds and the white spruce chloroplast genome. The dot plot was generated using the nucmer, show-coord, delta-filter and mummerplot utilities of MUMmer (v3.23; [31]). The x axis shows the position on the white spruce chloroplast genome in base pairs (bp).
(PDF)

S4 Fig **Alignments of a previously published Sitka spruce chloroplast genome (GenBank accession EU998739.3) to (A) our Sitka spruce chloroplast genome (“Sitka”) and (B) *P*. *thunbergii* (GenBank accession NC_001631).** The cross_match alignments (v1.080721; http://www.phrap.org) were visualized using XMatchView (v0.2; http://www.bcgsc.ca/platform/bioinfo/software/xmatchview). The histogram at the top of the figures shows the sequence identity over the length of the alignment, including those from repeated sequences. The dark blue represents sequences repeated once, while the lighter blue represents sequences repeated twice. The lower sections represent co-linear and inverted sequence alignment blocks in blue and pink, respectively.
(PDF)

S1 Table Summary of read subsets based on GemCode index multiplicity. Read subsets are based on the number associated reads for each index.
(DOCX)

S2 Table Results of QUAST analysis. QUAST (v3.1; [12]) was run on the contigs (> = 500bp) from the ABySS assembly and the scaffolds resulting from the LINKS runs using these contigs. The white spruce chloroplast genome was used as the reference genome.
(DOCX)

S3 Table Failure-mode analysis of Sealer gap-filling runs on the Sitka spruce chloroplast scaffold. Failure breakdown shown for the lower k-mer sized used (k = 35).
(DOCX)

S4 Table Best BLASTX hits of the 14 open reading frames (ORFs) to the NCBI-nr protein database. Four ORFs (orf5,8,10,12) have hits to annotated genes. Nine ORFs hit open reading frames (orf1,2,3,4,6,7,9,11,14). One ORF has no significant hits (orf13). Excluding it, every other ORF has a high-scoring hit to either a Pinus or Picea species, even though it may not necessarily be the best hit (not shown).
(DOCX)

S1 File Conifer chloroplast genomes nucleotide frequencies. Positional breakdown of nucleotide frequencies for five chloroplast genome sequences using MEGA7 [22].
(XLS)
