# Supplementary material for: Assembly of the Complete Sitka Spruce Chloroplast Genome Using 10X Genomics’ GemCode Sequencing Data
Source: PLoS One. 2016 Sep 15;11(9):e0163059. doi: 10.1371/journal.pone.0163059 (PMC5025161; doi:10.1371/journal.pone.0163059)
Supplement: S1 Fig — High-frequency GemCode read bins (> = 5k) were assembled with ABySS [11], contigs scaffolded with LINKS [13] and assembly gaps filled with Sealer [14] until all unknown bases (Ns) resolved. The genome completed by extending the ends with Konnector [17]. (PDF) [file pone.0163059.s001.pdf]

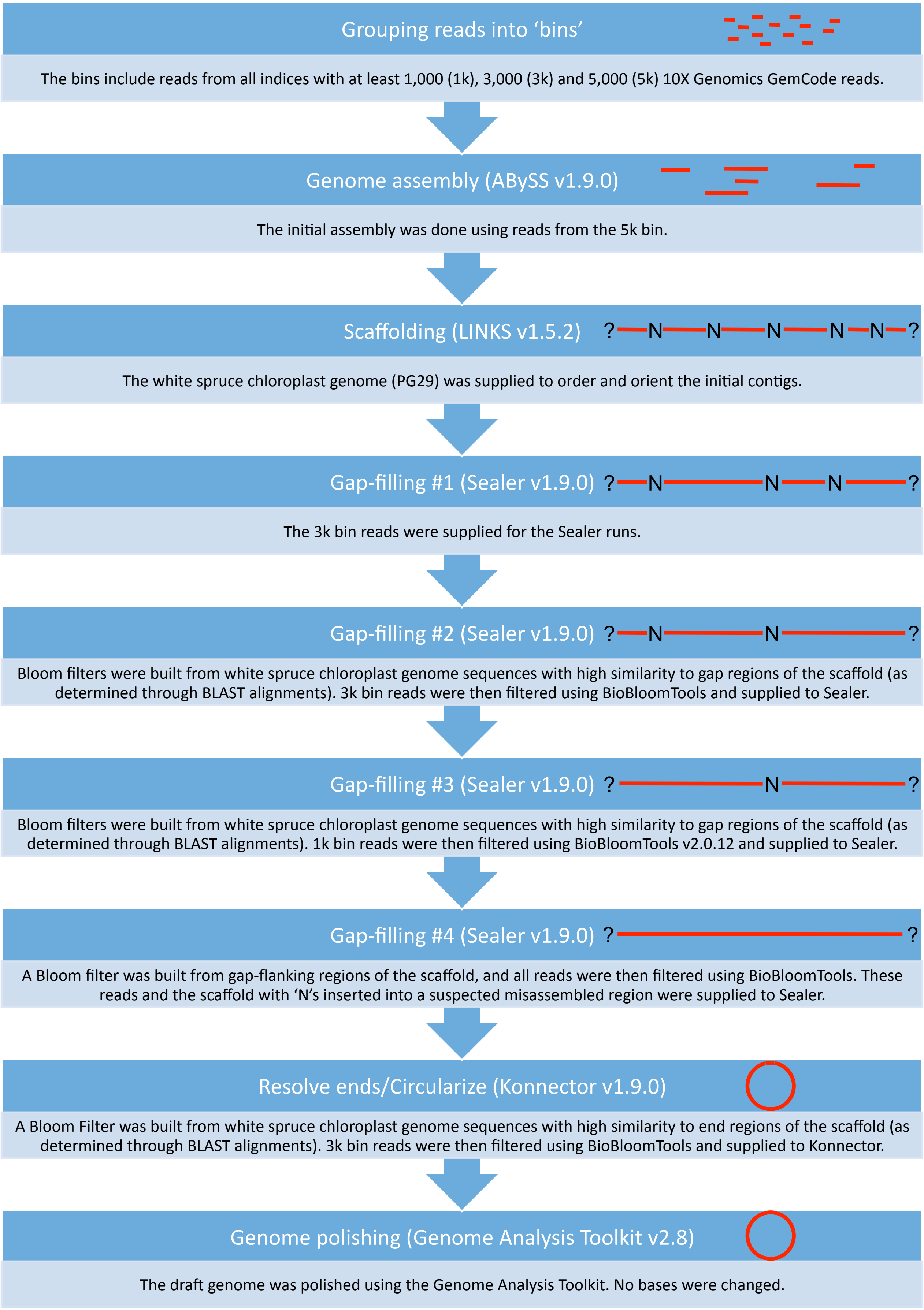

S1 Fig. Sitka spruce chloroplast genome assembly and finishing strategy. High-frequency GemCode reads (>=5k) were assembled with ABySS [11], contigs scaffolded with LINKS [13] and assembly gaps filled with Sealer [14] until all unknown bases (Ns) resolved. The genome completed by extending the ends with Konnector [17].
