# Supplementary material for: Assembly of the Complete Sitka Spruce Chloroplast Genome Using 10X Genomics’ GemCode Sequencing Data
Source: PLoS One. 2016 Sep 15;11(9):e0163059. doi: 10.1371/journal.pone.0163059 (PMC5025161; doi:10.1371/journal.pone.0163059)
Supplement: S2 Fig — Indices are in ascending order with respect to the number of associated reads, and only fully sequenced indices (containing no ambiguous bases) are included. (PDF) [file pone.0163059.s002.pdf]

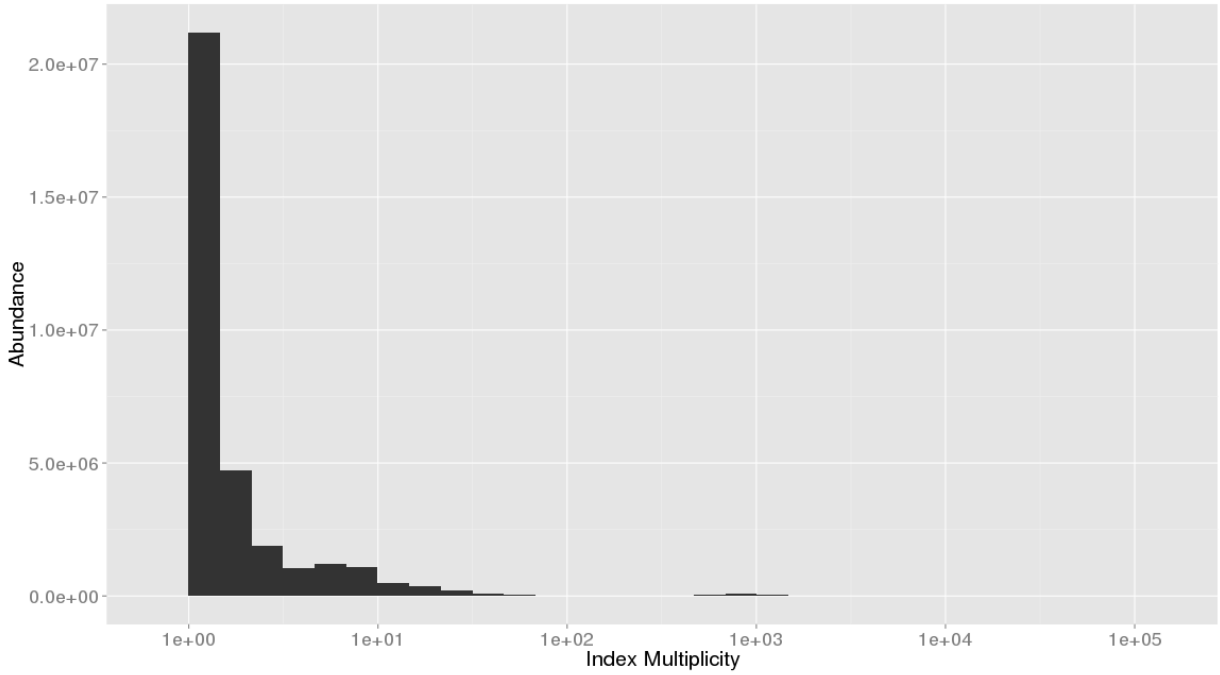

S2 Fig. Distribution of GemCode index multiplicity. Indices are in ascending order with respect to the number of associated reads, and only fully sequenced indices (containing no ambiguous bases) are included.
