# Supplementary material for: Assembly of the Complete Sitka Spruce Chloroplast Genome Using 10X Genomics’ GemCode Sequencing Data
Source: PLoS One. 2016 Sep 15;11(9):e0163059. doi: 10.1371/journal.pone.0163059 (PMC5025161; doi:10.1371/journal.pone.0163059)
Supplement: S3 Fig — The dot plot was generated using the nucmer, show-coord, delta-filter and mummerplot utilities of MUMmer (v3.23; [31]). The x axis shows the position on the white spruce chloroplast genome in base pairs (bp). (PDF) [file pone.0163059.s003.pdf]

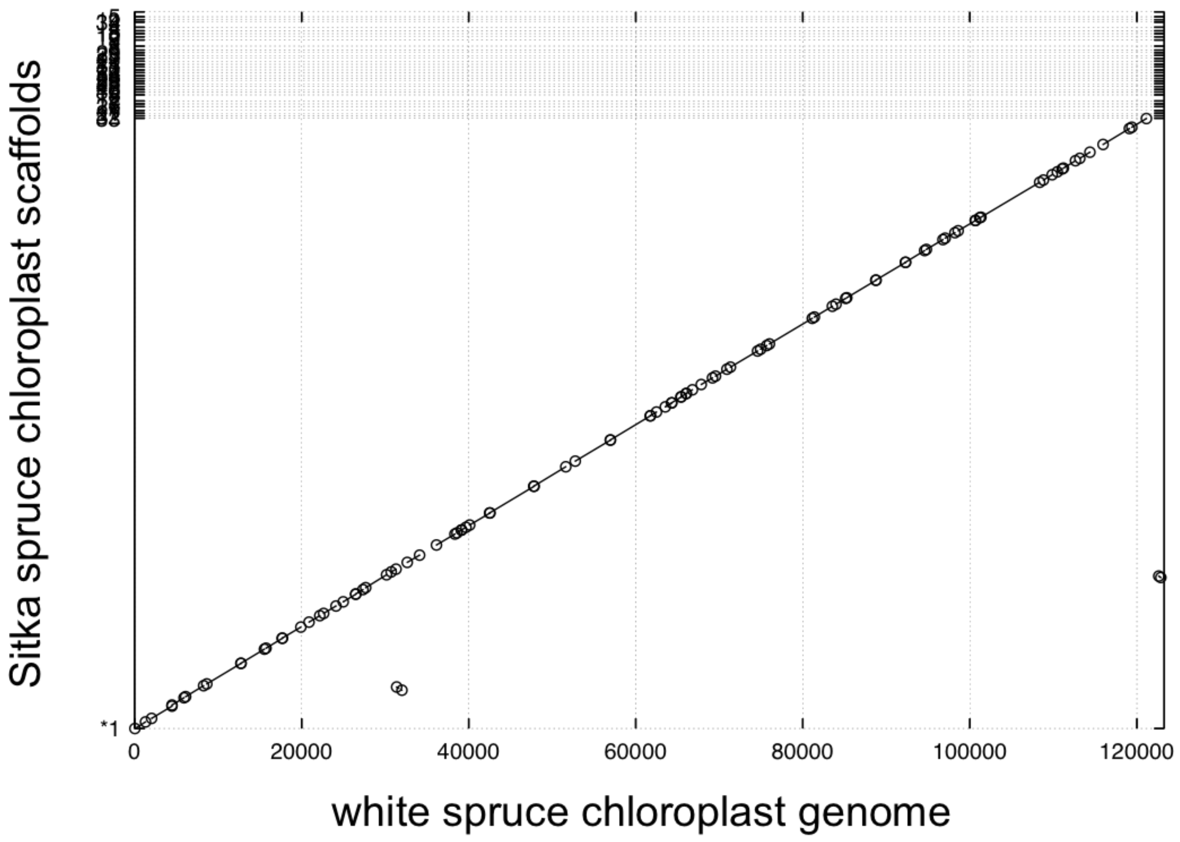

S3 Fig. Dot plot of alignment between the post-LINKS Sitka spruce chloroplast scaffolds and the white spruce chloroplast genome. The dot plot was generated using the nucmer, show-coord, delta-filter and mummerplot utilities of MUMmer (v3.23 [31]). The x axis shows the position on the white spruce chloroplast genome in base pairs (bp).
