# Supplementary material for: Assembly of the Complete Sitka Spruce Chloroplast Genome Using 10X Genomics’ GemCode Sequencing Data
Source: PLoS One. 2016 Sep 15;11(9):e0163059. doi: 10.1371/journal.pone.0163059 (PMC5025161; doi:10.1371/journal.pone.0163059)
Supplement: S4 Fig — Alignments of a previously published Sitka spruce chloroplast genome (GenBank accession EU998739.3) to (A) our Sitka spruce chloroplast genome (“Sitka”) and (B) P. thunbergii (GenBank accession NC_001631). The cross_match alignments (v1.080721; http://www.phrap.org) were visualized using XMatchView (v0.2; http://www.bcgsc.ca/platform/bioinfo/software/xmatchview). The histogram at the top of the figures shows the sequence identity over the length of the alignment, including those from repeated sequences. The dark blue represents sequences repeated once, while the lighter blue represents sequences repeated twice. The lower sections represent co-linear and inverted sequence alignment blocks in blue and pink, respectively. (PDF) [file pone.0163059.s004.pdf]

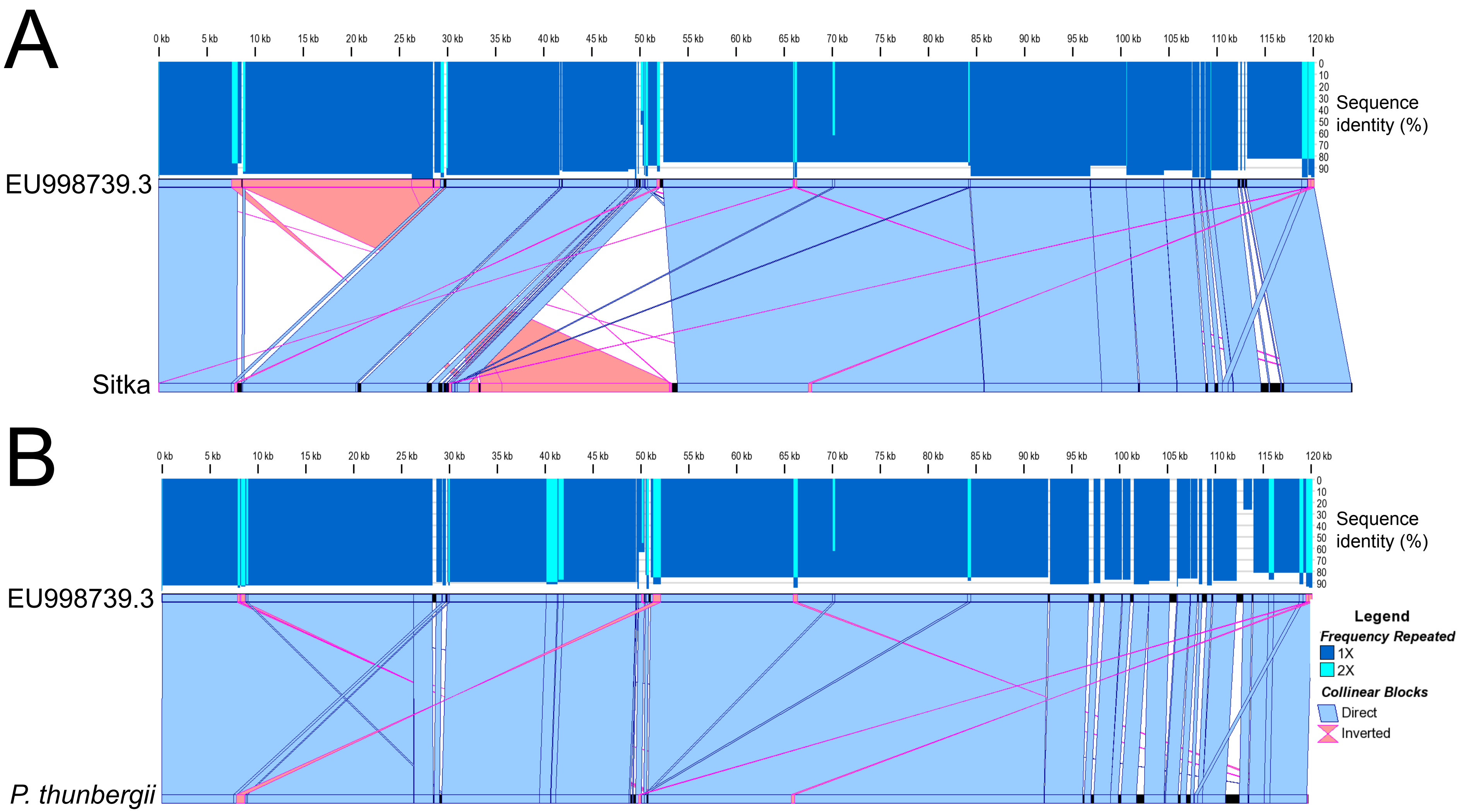

S4 Fig. Alignments of a previously published Sitka spruce chloroplast genome (GenBank accession EU998739.3) to (A) our Sitka spruce chloroplast genome ("Sitka") and (B) *P. thunbergii* (GenBank accession NC_001631). The cross_match alignments (v1.080721; http://www.phrap.org) were visualized using XMatchView (v0.2; http://www.bcgsc.ca/platform/bioinfo/software/xmatchview). The histogram at the top of the figures shows the sequence identity over the length of the alignment, including those from repeated sequences. The dark blue represents sequences repeated once, while the lighter blue represents sequences repeated twice. The lower sections represent co-linear and inverted sequence alignment blocks in blue and pink, respectively.
