# Supplementary material for: Assembly of the Complete Sitka Spruce Chloroplast Genome Using 10X Genomics’ GemCode Sequencing Data
Source: PLoS One. 2016 Sep 15;11(9):e0163059. doi: 10.1371/journal.pone.0163059 (PMC5025161; doi:10.1371/journal.pone.0163059)
Supplement: S1 Table — Read subsets are based on the number associated reads for each index. (DOCX) [file pone.0163059.s005.docx]

**S1 Table. Summary of read subsets based on GemCode index multiplicity.** Read subsets are based on the number associated reads for each index.

| **Number of reads per index** | **Raw reads** | **>=1** | **>=1,000** | **>=3,000** | **>=5,000** |
| --- | --- | --- | --- | --- | --- |
| **Number of indices** | 33,754,686*^ | 32,687,880 | 65,351 | 1,580 | 238 |
| **Total number of read pairs** | 294,085,217 | 290,367,368 | 99,464,078 | 7,062,581 | 2,290,669 |
| **Percent of total reads** | 100.00% | 98.74% | 33.82% | 2.40% | 0.78% |
| **Average Number of read pairs per index** | 8.71 | 8.88 | 1,522.00 | 4,469.99 | 9,624.66 |

***For the raw reads, all indices (including those with N’s) are included in the count. For all other read sets, only the indices without N’s were used for the binning.**

**^The total number of indices is greater than the number of unique indices introduced in the GemCode library preparation stage (750,000) due to sequencing errors.**
