# Supplementary material for: Assembly of the Complete Sitka Spruce Chloroplast Genome Using 10X Genomics’ GemCode Sequencing Data
Source: PLoS One. 2016 Sep 15;11(9):e0163059. doi: 10.1371/journal.pone.0163059 (PMC5025161; doi:10.1371/journal.pone.0163059)
Supplement: S2 Table — QUAST (v3.1; [12]) was run on the contigs (> = 500bp) from the ABySS assembly and the scaffolds resulting from the LINKS runs using these contigs. The white spruce chloroplast genome was used as the reference genome. (DOCX) [file pone.0163059.s006.docx]

**S2 Table. Results of QUAST analysis.** QUAST (v.3.1; [12]; default parameters) was run on the contigs (>= 500bp) from the ABySS assembly and the scaffolds resulting from the LINKS runs using these contigs. The white spruce chloroplast genome was used as the reference genome.

| **Query Sequence** | **Post-ABySS contigs >= 500bp** | **Post-LINKS scaffolds** |
| --- | --- | --- |
| **Number of contigs/scaffolds** | 108 | 34 |
| **Number of misassemblies** | 0 | 0 |
| **Number of unaligned contigs/scaffolds** | 35 | 33 |
| **Number of aligned contigs/scaffolds** | 73 | 1 |
| **Number of N’s** | 0 | 18,458 |
| **Genome fraction** | 81.09% | 80.32% |
| **N50** | 1,387 | 122,565 |
| **NA50** | 1,387 | 100,249 |
