# Supplementary material for: Assembly of the Complete Sitka Spruce Chloroplast Genome Using 10X Genomics’ GemCode Sequencing Data
Source: PLoS One. 2016 Sep 15;11(9):e0163059. doi: 10.1371/journal.pone.0163059 (PMC5025161; doi:10.1371/journal.pone.0163059)
Supplement: S3 Table — Failure breakdown shown for the lower k-mer sized used (k = 35). (DOCX) [file pone.0163059.s007.docx]

**S3 Table. Failure-mode analysis of Sealer gap-filling runs on the Sitka spruce chloroplast scaffold.** Failure breakdown shown for the lower k-mer sized used (k=35).

| **Breakdown of reasons for failure to fill the gaps** | **Number of gaps** |
| --- | --- |
| No start/goal kmer | 0 |
| No path | 0 |
| Too many paths (>20) | 1 |
| Too many branches (>3000) | 5 |
| Too many path/path mismatches | 0 |
| Too many path/read mismatches | 0 |
| Contains cycle | 0 |
| Exceeded memory limit | 0 |
| **TOTAL NUMBER OF UNFILLED GAPS** | **6** |
