# Supplementary material for: Assembly of the Complete Sitka Spruce Chloroplast Genome Using 10X Genomics’ GemCode Sequencing Data
Source: PLoS One. 2016 Sep 15;11(9):e0163059. doi: 10.1371/journal.pone.0163059 (PMC5025161; doi:10.1371/journal.pone.0163059)
Supplement: S4 Table — Four ORFs (orf5,8,10,12) have hits to annotated genes. Nine ORFs hit open reading frames (orf1,2,3,4,6,7,9,11,14). One ORF has no significant hits (orf13). Excluding it, every other ORF has a high-scoring hit to either a Pinus or Picea species, even though it may not necessarily be the best hit (not shown). (DOCX) [file pone.0163059.s008.docx]

**S4 Table. Best BLASTX hits of the 14 open reading frames (ORFs) to the NCBI-nr protein database.** Four ORFs (orf5,8,10,12) have hits to annotated genes. Nine ORFs hit open reading frames (orf1,2,3,4,6,7,9,11,14). One ORF has no significant hits (orf13). Excluding it, every other ORF has a high-scoring hit to either a *Pinus* or *Picea* species, even though it may not necessarily be the best hit (not shown).

| **ORF** | **Start** | **End** | **Strand** | **Length (bp)** | **Best hit** | **Gene** | **Species** | **BLASTX**  **Score** | **E value** |
| --- | --- | --- | --- | --- | --- | --- | --- | --- | --- |
| orf1 | 4398 | 4523 | + | 126 | ref\|YP_001152057.1 | ORF41a | *Pinus koraiensis* | 79.3 | 5e-19 |
| orf2 | 14197 | 14526 | + | 330 | dbj\|BAA04352.1 | ORF42a | *Pinus thunbergii* | 66.2 | 1e-12 |
| orf3 | 20645 | 20848 | + | 204 | ref\|NP_042408.1 | ORF119 | *Pinus thunbergii* | 36.6 | 0.47 |
| orf4 | 28478 | 28681 | + | 204 | ref\|NP_817199.1 | ORF67e | *Pinus koraiensis* | 85.1 | 2e-20 |
| orf5 | 30837 | 31148 | + | 312 | gb\|AAP80884.1 | rps4 | *Picea smithiana* | 154 | 6e-46 |
| orf6 | 35749 | 35904 | - | 156 | ref\|YP_001152092.1 | ORF100 | *Pinus koraiensis* | 77.8 | 1e-17 |
| orf7 | 53589 | 53807 | - | 219 | gb\|ACN39997.1 | unknown | *Picea sitchensis* | 76.3 | 7e-17 |
| orf8 | 68781 | 69188 | + | 408 | ref\|YP_009158607.1 | ndhK | *Encephalartos lehmannii* | 131 | 3e-35 |
| orf9 | 85089 | 85292 | - | 204 | ref\|NP_042472.1 | ORF77 | *Pinus thunbergii* | 78.2 | 1e-17 |
| orf10 | 110595 | 111131 | - | 537 | ref\|YP_009232295.1 | ycf2 | *Picea jezoensis* | 335 | 1e-103 |
| orf11 | 112748 | 112930 | + | 183 | gb\|KJB79837.1 | hypothetical | *Gossypium raimondii* | 49.3 | 6e-06 |
| orf12 | 115029 | 115187 | + | 159 | gb\|AKJ77598.1 | ndhB | *Dioscorea nipponica* | 85.9 | 4e-19 |
| orf13 | 115241 | 115528 | + | 288 | No hits found | NA | NA | NA | NA |
| orf14 | 116390 | 116575 | + | 186 | ref\|YP_001152258.1 | ORF40z | *Pinus koraiensis* | 32.7 | 3.7 |
